# LYMPHOPARIETAL INDEX IN ESOPHAGEAL CANCER IS STRONGER THAN TNM STAGING IN LONG-TERM SURVIVAL PROGNOSIS IN A LATIN-AMERICAN COUNTRY

**DOI:** 10.1590/0102-672020200003e1547

**Published:** 2021-01-15

**Authors:** Manuel FIGUEROA-GIRALT, Catalina VALENZUELA, Andrés TORREALBA, Attila CSENDES, Italo BRAGHETTO, Enrique LANZARINI, Maher MUSLEH, Owen KORN, Hector VALLADARES, Solange CORTÉS

**Affiliations:** 1 Department of Surgery, Universidad de Chile Clinical Hospital, Santiago, Chile.

**Keywords:** Esophageal neoplasms, Survival, Prognosis, Neoplasias esofágicas, Sobrevida, Pronostico

## Abstract

**Background:**

The identification of prognostic factors of esophageal cancer has allowed to predict the evolution of patients.

**Aim:**

Assess different prognostic factors of long-term survival of esophageal cancer and evaluate a new prognostic factor of long-term survival called lymphoparietal index (N+/T).

**Method:**

Prospective study of the Universidad de Chile Clinical Hospital, between January 2004 and December 2013. Included all esophageal cancer surgeries with curative intent and cervical anastomosis. Exclusion criteria included: stage 4 cancers, R1 resections, palliative procedures and emergency surgeries.

**Results:**

Fifty-eight patients were included, 62.1% were men, the average age was 63.3 years. A total of 48.3% were squamous, 88% were advanced cancers, the average lymph node harvest was 17.1. Post-operative surgical morbidity was 75%, with a 17.2% of reoperations and 3.4% of mortality. The average overall survival was 41.3 months, the 3-year survival was 31%. Multivariate analysis of the prognostic factors showed that significant variables were anterior mediastinal ascent (p=0.01, OR: 6.7 [1.43-31.6]), anastomotic fistula (p=0.03, OR: 0.21 [0.05-0.87]), N classification (p=0.02, OR: 3.8 [1.16-12.73]), TNM stage (p=0.04, OR: 2.8 [1.01-9.26]), and lymphoparietal index (p=0.04, RR: 3.9 [1.01-15.17]. The ROC curves of lymphoparietal index, N classification and TNM stage have areas under the curve of 0.71, 0.63 and 0.64 respectively, with significant statistical difference (p=0.01).

**Conclusion:**

The independent prognostic factors of long-term survival in esophageal cancer are anterior mediastinal ascent, anastomotic fistula, N classification, TNM stage and lymphoparietal index. In esophageal cancer the new lymphoparietal index is stronger than TNM stage in long-term survival prognosis.

## RESUMO

**Racional:**

A identificação de fatores prognósticos do câncer de esôfago permitiu prever a evolução dos pacientes.

**Objetivo:**

Avaliar diferentes fatores prognósticos da sobrevida em longo prazo do câncer de esôfago e avaliar um novo fator prognóstico da sobrevida em longo prazo chamado índice linfoparietal (N+/T).

**Método:**

Estudo prospectivo do Hospital Clínico da Universidade do Chile, entre janeiro de 2004 e dezembro de 2013. Incluiu todas as operações de câncer de esôfago com intenção curativa e anastomose cervical. Os critérios de exclusão incluíram: câncer em estágio 4, ressecções R1, procedimentos paliativos e operações de emergência.

**Resultados:**

Cinquenta e oito pacientes foram incluídos, 62,1% eram homens, a idade média foi de 63,3 anos. Um total de 48,3% eram escamosos, 88% eram cânceres avançados, a colheita média de linfonodos foi de 17,1. A morbidade cirúrgica pós-operatória foi de 75%, com 17,2% de reoperações e 3,4% de mortalidade. A sobrevida global média foi de 41,3 meses, a sobrevida em três anos foi de 31%. A análise multivariada dos fatores prognósticos mostrou que variáveis significativas foram elevação pelo mediastinal anterior (p=0,01, OR: 6,7 [1,43-31,6]), fístula anastomótica (p=0,03, OR: 0,21 [0,05-0,87]), classificação N (p=0,02, OR: 3,8 [1,16-12,73]), estágio TNM (p=0,04, OR: 2,8 [1,01-9,26]) e índice linfoparietal (p=0,04, RR: 3,9 [1,01-15,17]. As curvas ROC do índice linfoparietal, classificação N e estádio TNM apresentam áreas abaixo da curva de 0,71, 0,63 e 0,64, respectivamente, com diferença estatística significativa (p=0,01).

**Conclusão:**

Os fatores prognósticos independentes de sobrevida em longo prazo no câncer de esôfago são a elevação mediastinal anterior, fístula anastomótica, classificação N, estágio TNM e índice linfoparietal. No câncer de esôfago, o novo índice linfoparietal é mais forte que o estágio TNM no prognóstico de sobrevida em longo prazo.

## INTRODUCTION

The identification of some prognostic factors in oncologic disease has allowed to predict patient’s evolution and guided therapeutic decision-making process to improve long-term survival^18^ ^,^ ^26^. However, in Chilean reality there are insufficient studies that analyze multiple prognostic factors of long-term survival in esophageal cancer^1^ ^-^ ^7^ ^,^ ^29^ ^-^ ^32^.

The main objective of this study was to assess different prognostic factors of long-term survival in esophageal cancer. Secondary objectives were: a) analyze post-operative evolution; b) determine global overall survival greater than three years (OS3); and c) assess the value of a new prognostic factor of long-term survival called lymphoparietal index (N+/T), previously validated in gastric cancer^12^ ^,^ ^13^.

## METHOD

This study was a prospective analysis of the oncological database of a Chilean University (Clinical Hospital of the University of Chile) between January 2004 and December 2013.

### Ethical standards

This article does not contain any experimental studies with human or animal subjects performed by any of the authors.

### Patients

All patients with esophageal cancer in adult population, surgically treated with a curative intent, were identified, and only total esophagectomies with gastric tube ascent and cervical anastomosis where included. All patients were presented to the hospital oncology committee and treated with neoadjuvant or adjuvant therapy according to tumor stage. Exclusion criteria included were: proximal tumors, Siewert 3, stage 4 cancers, R1 resections, palliative procedures and emergency surgeries

### Surgical technique.

The surgeries were performed by surgeons with vast experience in oncological esophagectomies. All patients were subjected to minimally invasive thoraco-abdominal esophagectomy and cervical anastomosis. The thoracic time was done in the first years transhiatal and then by videothoracoscopy in left lateral decubitus. The gastric tube was made in the first years open and then laparoscopic with linear staplers from the distal aspect of the lesser curvature to the gastric fundus, 5 cm to the grater curve of the stomach preserving the gastro-omental arcade. The left gastro-omental vessels, right and left gastric vessels were cut. The gastric tube was pulled upwards to the cervical compartment through anterior or posterior mediastinal way according to surgeon preference. The lymphadenectomy was standard in two fields. All patients had an intra-operative contemporary biopsy. 

### Definitions

The definitions used were: a )TNM classification was standarized using the AJCC 7th edition^5^; b) the lymphoparietal index (N+/T) calculates the quotient between the number of lymph nodes that are positive for adenocarcinoma metastasis and the T classification of the patient^12^ ^,^ ^13^, examples: 1/T1a=1/1=1, 6/T3 =6/3=2, 24/T4b=24/4=6) and the ratio results were divided into N+/T_A_: 0-0.5 and N+/T_B_: >0.5; c) surgical mortality was defined as occurring from the moment of surgery up to postoperative day 90; d) global survival was defined as of when the patient was discharged from the hospital, eliminating surgical mortality; e) long term survival was defined as survival greater than three years postoperative; f) zero time for determining prognostic association was the esophagectomy.

### Follow up

The present study had 100% follow up. The database was completed in a prospective manner: the survival update was carried out annually using the database of our hospital and the Chilean Civil Registry. 

### Statistical analysis

The prognostics factors evaluated were demographic, clinical, surgical, anatomopathological and prognostic indexes, 31 variables in total. The distribution of variables was determined by the Shapiro-Wilk test. In accordance with this test, the continuous variables with parametric distribution (ordinal) were expressed on average and standard deviation (SD), while for the non-parametric distribution (nominal) the median and inter-quartile (IC_25%-75%_) ranges were used. The categorical variables were described in percentages. The Fisher, x^2^, t Student and Wilcoxon Rank-Sum tests were used based on the characteristics and distribution of the variables. For the analytical statistical analysis, the Stata^R^ 14 program was used and p< 0.05 was considered statistically significant. Univariate and multivariate analyses were performed calculating the odds ratio (OR) with a 95% confidence interval (CI). The Kaplan-Meier method was used to calculate the survival curves, and the ROC curves to assess the prognosis accuracy of the variables^14.^

## RESULTS

A total of 95 patients had surgery for esophageal cancer and 55 were included in the study according to exclusion criteria. The mean age was 63.3 years (+10.4 DS) of which 62.1% were male, 74.1% of patients presented co-morbidities with tabacco, high blood pressure and pathological gastroesophageal reflux disease being the most common with 48.3%, 44.83% and 43.1% respectively. According to the ASA classification, 52.7% were ASA I, 47.3% were ASA II and III. 

With regards to the clinical manner, 81.8% presented epigastric pain, 50.9% weight loss and 21.8% pain. Anemia (hematocrit <35%) was observed in 16.4%, while protein malnutrition (albumin <3.5 mg/dl) was present in 7.3%.

In reference to the surgical technique, 61.8% of patients had anterior mediastinal pull-up of gastric tube. The median global lymph node harvest was 17.1 lymph nodes (IC_25-75%_: 11-35). 

The mean hospital stay was 24 days (+18 DS). Postoperative morbidity corresponded to 75%, reoperations to 17.2%, while surgical mortality was 3.4% (Table 1).

The histopathological study revealed that 65.5% of the tumors were localized in the distal esophagus, 52.7% of the sample was adenocarcinoma, 88% of the tumor were advanced and 72.7% of all had moderate to poor degree of differentiation. The TNM stage is specified in Table 2.

**FIGURE 1:**
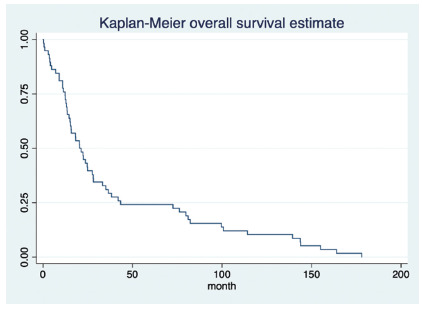
Estimated overall survival of the cohort

The mean global survival was 41.3 months (interval between 1 and 178 months, DS +/- 47.2). The rate of patients with an OS3 was 32.7%. The survival curve is detailed in Figure 1. 

**FIGURE 2:**
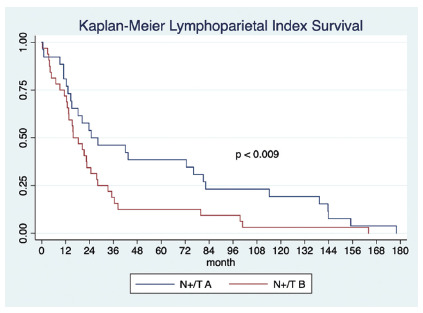
Overall survival analysis according to lymphoparietal index subgroups N+/TA (0-0.5) and N+/TB (>0.5)

In the lymphoparietal index Kaplan-Meier analysis, a statistically significant difference was seen in the global long-term survival between subgroups (N+/T_A_ and N+/T_B_) p<0.009, Figure 2).

The multivariate analysis of the prognostic factors is represented in Table 2, the significant variables are: anterior mediastinal pull-up, anastomotic fistula, N classification, TNM stage, and lymphoparietal index (Table 2).

**FIGURE 3:**
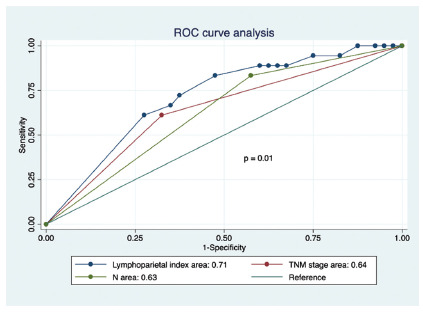
ROC curve analysis according to overall survival

The ROC curve of lymphoparietal index, N classification and TNM stage showed the respectively areas below the curves 0.71, 0.63 and 0.64 (p=0.01, Figure 3)

**TABLE 1:** Univariable analysis of demographic, clinical, surgical and oncologic variables of long-term survival in esophageal cancer.

Variável	OS< 3		OS >3		Univariable analysis		
	n=37	%	n=18	%	p	OR	CI 95%
Gender							
Male	26	70.3%	8	44.4%	0.08		
Female	11	29.7%	10	55.6%			
Age	63.5		61.7		0.17		
ASA							
I	22	59.5%	7	38.9%	0.07		
II-III	15	40.5%	11	0.0%			
Comorbidities							
Hypertension	19	51.4%	5	27.8%	0.14		
Diabetes	7	18.9%	1	5.6%	0.25		
COPD	4	10.8%	0	0.0%	0.29		
Tabacco	19	51.4%	8	44.4%	0.7		
BMI (kg/mt2)							
<25	15	40.5%	7	38.9%	0.99		
>25	22	59.5%	11	61.1%			
Esophageal disease							
GERD	13	35.1%	11	61.1%	0.08		
BARRETT	8	21.6%	7	38.9%	0.21		
HH	6	16.2%	1	5.6%	0.41		
Symptoms and signs							
Disphagia	30	81.1%	15	83.3%	>0.99		
Weight loss	22	59.5%	6	33.3%	0.08		
Pain	9	24.3%	3	16.7%	0.72		
Laboratory							
HTO <35%	8	21.6%	1	5.6%	0.24		
Alb <3.5mg/dl	2	5.4%	2	11.1%	0.59		
Localization							
Middle	11	29.7%	8	44.4%	0.37		
Distal	26	70.3%	10	55.6%			
Pull-up							
AP	20	54.1%	14	77.8%	0.04	2.02	0.002-0.48
PP	17	45.9%	4	22.2%			
Esophageal fistula							
Yes	29	78.4%	8	44.4%	0.016	1.76	1.11-3.25
No	8	21.6%	10	55.6%			
Mediastinal abscess							
Yes	7	18.9%	3	16.7%	>0.99		
No	30	81.1%	15	83.3%			
Pleural effusion							
Yes	2	5.4%	2	11.1%	0.59		
No	35	94.6%	16	88.9%			
Pneumonia							
Yes	8	21.6%	1	5.6%	0.24		
No	29	78.4%	17	94.4%			
Arrythmia							
Yes	5	13.5%	2	11.1%	>0.99		
No	32	86.5%	15	83.3%			
Histology							
EC	18	48.6%	8	44.4%	>0.99		
ADN	19	51.4%	10	50.0%			
Tumor grade							
Well	9	24.3%	6	33.3%	0.02	0.6	0.35-0.89
Moderate	18	48.6%	12	66.7%			
Bad	10	27.0%	0	0.0%			
TNM							
Tis	0	0.0%	1	5.6%	0.06		
T1a	0	0.0%	1	5.6%			
T1b	2	5.4%	2	11.1%			
T2	9	24.3%	5	27.8%			
T3	26	70.3%	7	38.9%			
N0	8	21.6%	11	61.1%	0.01	0.54	0.29-0.86
N1	11	29.7%	4	22.2%			
N2	10	27.0%	4	22.2%			
N3	7	18.9%	0	0.0%			
Stage							
0	0	0.0%	1	5.6%	0.023	0.00	0.00-0.87
IB	0	0.0%	2	11.1%			
IIA	4	10.8%	7	38.9%			
IIB	5	13.5%	1	5.6%			
IIIA	4	10.8%	1	5.6%			
IIIB	9	24.3%	6	33.3%			
IVA	7	18.9%	0	0.0%			
Lymphoraietal index							
A (0-0.5)	13	35.1%	13	72.2%	0.02	0.6	0.37-0.88
B (>0.5)	24	64.9%	5	27.8%			

**TABLE 2:** Multivariable analysis of long-term survival in esophageal cancer

Variable	Multivariable analysis		
	p	OR	CI 95%
Gender	0.03	3.9	1.10-14.14
Pull-up	0.01	6.7	1.43-31.60
Fistula	0.03	0.2	0.05-0.87
N	0.02	3.8	1.16-12.73
TNM stage	0.04	2.8	1.01-9.26
Lymphoparietal index	0.04	3.9	1.01-15.17

## DISCUSSION

The main results of this study suggest the following: 1) the Chilean esophageal cancer is experimenting an epidemiological transition; 2) there are different variables that significantly predict the population susceptible to achieving postoperative long-term survival; 3) the lymphoparietal index is as accurate as TNM system for predicting survival more than three years in patients who underwent surgery for esophageal carcinoma with curative intent. 

The esophageal cancer epidemiology has changed over the past 40 years. In our country, comparing previous reports to the present results: the location of the tumor in the lower esophagus has increased from 26% to 65%, the adenocarcinoma increased from 14% to 52%, and the surgical mortality dropped from 6% to 3%^3^ ^,^ ^7^ ^,^ ^29^. This changes probably are associated to the increase of Barrett´s esophagus in de GERD secondary to overweight that in the last national surveillance program reaches 70%^11^.

The SVg3 of the patients in this study was 32.7%, which is very similar to previous national reports^4^ ^,^ ^7^ ^,^ ^29^, but lower than other international ones^25^ ^,^ ^27^ ^,^ ^28^. Some explanations for this numbers are: a) long period of study with worst results in the first years; b) high incidence of advanced disease in our cohort; and c) small sample size due to the low incidence of this pathology in our country that bias the impact of new advances in neoadjuvant and adjuvant therapy.

The prognostic effectiveness of the TNM classification to guide therapeutics is well known^18^. Recently, different complementary prediction factors of long-term survival have been described. 

### Gender

The results of this publication suggest an independent association between females and long-term survival with p=0.03 (OR: 3.9). This finding has been study by other groups, suggesting a possible estrogen protective effect, especially in adenocarcinoma^17^ but also squamous cell carcinoma^21^.

### Age

The role of age in the prognosis of patients subjected to oncologic procedures has been studied many foregut cancers, being gastric^8^ ^,^ ^12^ and esophageal cancer^20^ ^,^ ^24^. These reports have demonstrated that older patients have an increased risk of surgical morbidity and lower long-term survival. These findings are not seen in the present study, which has been documented in other series as well^27^.

### Nutritional state

The nutritional state has been studied by different authors in the preoperative and postoperative stages. 

In a retrospective Brazilian study, Marin^16^ showed that lower BMI, lymphocytes and albumin, where associated with greater risk of infectious surgical complications and mortality, although no multivariable analysis was performed. 

In a recent retrospective Japan study, Schichinohe^22^ demonstrated that not only BMI and cross-sectional area of the psoas muscle index, but also an index between these two variables were independent factors associated with higher risk of anastomosis leaks and 3-years overall survival.

In our study there was no independent correlation between BMI, weight loss, neither albumin level to OS3, which has been concluded by other as well^14^. 

### Circulating tumor cells

Measurement of circulating tumor cells (CTC) and its prognosis, has been study in different solid tumors including esophageal cancer^19^. Recently, a Chinese prospective study analyzed the levels of CTC in squamous cell esophageal carcinoma measured pre and post-surgery. The results showed that a change in CTC between first diagnosis and 13 days after surgery of >2/7.5 ml peripheral blood, is associated with lower progression-free-survival^35^. 

### Localization, tumor grade and TNM

Classically, tumor localization and grade of differentiation are associated with lower long-term survival. The previous actualization of AJCC guideline for esophageal cancer, allowed to differentiate between different subtypes according to localization and tumor grade^18^. 

Interestingly, in a retrospective Chinese analysis of 302 esophageal carcinoma staged T3N0M0, Situ et al^24^, concluded that localization and tumor grade didn´t have an independent influence on patient survival, this is supported by other study^10^ ^,^ ^15^. However, in a different analysis, with the same objective but in T2N0M0 patients, tumor grade shows to be an independent factor, whereas localization wasn´t ^23^. 

Other publications have compared 6^th^ vs. 7^th^ TNM staging, concluding that 7^th^ edition is more accurate than 6^th^ in terms of prognosis^15^. 

In our cohort neither the localization nor tumor grade affected long-term survival, while TNM staging was independent prognostic factors. 

### Route of pull up and anastomotic fistula

The anterior (AP) or posterior mediastinal pull-up (PP) dilemma, has been analyzed in different series, there has been even combinations of this techniques from posterior to anterior mediastinum after esophagectomy^34^. 

Classically AP have had more leakages, lower Clavien-Dindo morbidity, and safer results if post-operative radiotherapy is required^2^ ^,^ ^9^.

Recent evidence with minimally invasive surgery supports no difference in lymph node harvested, ICU and hospital stay, postoperative morbidity, and in-hospital mortality^33^. 

A previous experience of our group showed similar rate of leaks for AP and PP (p>0,05), but a worst post-operative morbidity concentrating all types CD III-V and lower OS3 for PP^5^. In the present study we found that AP is an independent prognostic factor for long term survival, probably because the lower rate of severe post-operative morbidity.

### Adjuvant therapy

Since CROSS study^28^, neoadjuvant chemo-radiation therapy is well stablished as a treatment standard in locally advance tumors with significant benefits. In our study we couldn´t include adjuvant therapy in the analysis, this is due the absence of registration in more than 20% of patients, the information bias of this under-registration, cannot make conclusion reliable in adjuvant therapy. This happens because some health provisional system in our country, can mandate an externalization of the service to another institution.

### Lymphoparietal index

Regarding the N+/T index, it has been validated in gastric cancer by our group^13^. The hypothesis is that lymph node metastatic potential of a tumor considering T classification could reliably predict patient prognosis and even be more accurate than TNM staging). In this study we found: a) lymphoparietal index is an independent prognostic factor (p=0.02, OR 3.9; CI 95% 1.01-15.17, Table 2); b) long-term survival probability is significant discriminated in both groups (N+/T_A_ vs. N+/T_B_; p=0.009, Figure 1); c) lymphoparietal index is comparable to TNM staging and even has better performance in OS3 prognosis (p=0.01, Figure 2).

The strengths of this investigation are the following: a) the analysis of the greatest number of prognostic variables for long-term survival for esophageal cancer reported in the domestic literature, and b) the provision of a new survival prediction index. The weaknesses are as follows: a) it covers a period of time in which there was a change in TNM classification, and treatment strategies, and b) it couldn`t include the adjuvant therapy used in the analysis. 

## CONCLUSION

The independent prognostic factors for more than three years survival in treatment of esophageal cancer in a Latin American country are: gender, anterior mediastinal pull-up, anastomotic fistula, N classification, TNM stage, and lymphoparietal index. Concomitantly, it has been able to provide a new prognostic quotient in the evaluation of esophageal carcinoma patients who have been resected with curative intent, the lymphoparietal index.
